# In vitro assessment of intra-operative and post-operative environment in reducing bladder cancer recurrence

**DOI:** 10.1038/s41598-021-04035-8

**Published:** 2022-01-07

**Authors:** Ryan Tsz-Hei Tse, Hongda Zhao, Christine Yim-Ping Wong, Angel Wing-Yan Kong, Ronald Cheong-Kin Chan, Ka-Fai To, Chi-Fai Ng, Jeremy Yuen-Chun Teoh

**Affiliations:** 1 Department of Surgery, S.H. Ho Urology Centre, Prince of Wales Hospital, The Chinese University of Hong Kong, 4/F LCW Clinical Sciences Building, 30-32 Ngan Shing Street, Shatin, New Territories, Hong Kong, China; 2 Department of Anatomical and Cellular Pathology, The Chinese University of Hong Kong, Hong Kong, China; 3 European Association of Urology-Young Academic Urologists (EAU-YAU) Urothelial Cancer Working Group, Amsterdam, The Netherlands

**Keywords:** Urogenital diseases, Bladder cancer, Urological cancer, Cancer, Cancer microenvironment

## Abstract

Urinary bladder cancer is a common cancer worldwide. Currently, the modality of treating and monitoring bladder cancer is wide. Nonetheless, the high recurrence rate of non-muscle-invasive bladder cancer after surgical resection is still unsatisfactory. Hereby, our study demonstrated whether the intra-operative and post-operative environments will affect bladder cancer recurrence utilizing in vitro cell line model. Bladder cancer cell lines were submerged in four different irrigating fluids for assessing their tumorigenic properties. Our results showed that sterile water performed the best in terms of the magnitude of cytotoxicity to cell lines. Besides, we also investigated cytotoxic effects of the four irrigating agents as well as mitomycin C (MMC) in normothermic and hyperthermic conditions. We observed that sterile water and MMC had an increased cytotoxic effect to bladder cancer cell lines in hyperthermic conditions. Altogether, our results could be translated into clinical practice in the future by manipulating the intra-operative and post-operative conditions in order to lower the chance of residual cancer cells reimplant onto the bladder, which in turns, reducing the recurrence rate of bladder cancers.

## Introduction

Urinary bladder cancer is the ninth most common cancer in the world. There are more than 550,000 new cases diagnosed and 220,000 deaths every year^1,2^. With the advancement in diagnostic techniques and nonsurgical therapies over the past decades, the treatment and surveillance of bladder cancer have been greatly improved. However, non-muscle-invasive bladder cancer (NMIBC) is still accompanied with high recurrence rate and the prognosis of muscle-invasive bladder cancer (MIBC) has also remained poor^3–5^ which in turns burdening the healthcare system.

Transurethral resection of bladder tumour (TURBT) is the current standard in the initial diagnosis and treatment of NMIBC. Conventionally, monopolar resection in non-conducting intra operative irrigation solutions such as 1.5% glycine and water, or bipolar resection in isotonic (0.9% NaCl) normal saline is commonly performed. Although TURBT is safe and relatively simple to perform, cancer cells can be released during the resection procedure. The floating tumour cells may reimplant to the bladder wall resulting in early disease recurrence^6^. After TURBT, intravesical instillations of chemotherapy such as Mitomycin-C (MMC) can be given to impose a cytotoxic effect on the floating tumour cells. Combining TURBT and an immediate post-operative instillation of chemotherapy can reduce the risk of recurrence by 35%^7^. In addition, synergism of hyperthermia and chemotherapy is suggested to impose a greater cytotoxic effect to residual cancer cells, therefore, further lower the chance of cancer recurrence after resection.

After resection, the tumour cells will be deprived of blood supply and cell death is an inevitable outcome unless early tumour reimplantation occurs. It was found that human bladder cancer cells form firm attachment onto the culture dish take place within one hour in vitro and reached maximum within 24 h, followed by proliferation of bladder cancer cells^8^. We believed the condition in vivo maybe similar as in vitro, therefore, it becomes extremely important to understand if the surrounding fluid environment has any beneficial or harmful effects on the floating tumour cells. A fluid environment which can avoid tumour reimplantation may be the key in avoiding tumour recurrence after a complete resection.

In this study, we investigated the effects of common irrigating fluids (including 1.5% glycine, normal saline and water) and mitomycin C, with or without hyperthermia, in terms of tumorigenic properties on four bladder cancer (CaB) cell lines. We believe the results may provide valuable insights on how we can manipulate the fluid environment in order to optimise the oncological outcomes.

## Material and methods

### Cell lines and reagents

Four human urinary bladder cancer cell lines (T24, UMUC3, RT4 and HTB9) were used in this study. T24 and RT4 cell lines were maintained in McCoy’s 5A Medium with high-glucose and L-glutamine (ThermoFisher Scientific Inc.). UMUC3 and HTB9 were grown in Minimum Essential Medium (MEM) (ThermoFisher Scientific Inc.) and in Roswell Park Memorial Institute (RPMI) 1640 Medium with L-glutamine (ThermoFisher Scientific Inc.) respectively. All the cells were stocked in liquid nitrogen in dimethyl sulfoxide (DMSO) for permanent storage until use. The cell experiments were performed within 40 passages of the cells. After trypsinization, 1 × 10^6^ T24, UMUC3, RT4 and HTB9 cells were prepared in suspension with sterile water, normal saline (Baxter), 1.5% glycine (Baxter), 10 µg/mL MMC (Sigma-Aldrich, Inc.), 100 µg/mL MMC, 1000 µg/mL MMC or culture media (as controls). Suspension cells in different reagents were incubated at 37 °C or 43 °C for 1 h. Cells were then centrifuged, and cell viability was assessed by 0.4% Trypan Blue dye exclusion test. Living cells were further tested by four different assays. All the assays were performed in triplicate and at least three independent experiments.

### Proliferation assay

The growth rates of T24, UMUC3, RT4 and HTB9 cells were tested by the proliferation assay. Living cells were first seeded in 96-well plates (100 µl/well) at a density of 1 × 10^4^ cells/mL. After seeding and incubation for 24 and 48 h, cells were treated with 10 µl 3-[4,5-dimethylthiazol-2-yl]-2,5-diphenyltetrazolium bromide (MTT) (ThermoFisher Scientific Inc.) and incubated for 4 h. MTT-mixture was removed and DMSO was added to dissolve the purple formazan produced by reduction of MTT by living cells and the absorbance was measured at 590 nm using a microplate reader.

### Adhesion assay

Adhesion assay was performed by plating 2 × 10^4^ cells/mL on Gibco^®^ Collagen I-coated plate (ThermoFisher Scientific Inc.). Cells were centrifuged at 71*g* for 5 min at room temperature (RT), followed by incubation for 15 min at 37 °C in a humidified incubator with 5% CO2. After incubation, media in each well containing cells was then removed and the whole plate underwent centrifugation upside down at 7*g* for 5 min at RT. A MTT assay was then performed. After incubation for 4 h, MTT-mixture was removed and DMSO was added, the absorbance at 590 nm was measured by a microplate reader.

### Invasion assay

Cell invasion assays were carried out using BD BioCoat Matrigel Invasion chambers (BD Biosciences, USA) according to manufacturer’s protocol. Briefly, 5 × 10^4^ cells/mL living cells were seeded in each transwell containing 0.5% serum medium. The transwell insert was plated onto the 24-well plated containing 5% serum medium as a lower compartment, serving as an attractant for the cells to invade from the upper compartment towards to lower compartment through the microporous Matrigel^®^ matrix-reconstituted membrane on the basement of each transwell insert. After incubation at 37 °C in a humidified incubator with 5% CO_2_ for 48 h, the transwell insert was removed and crystal violet stain was performed, accumulating the cell nuclei of the invasive cells which could enzymatically degrade the Matrigel^®^ matrix and invade through membrane pores to the lower compartment. Stained cells were photographed by an inverted microscope and counted by the Image J software in three randomly selected fields.

### Colony formation assay

To assess the cell’s ability to grow into a colony, 1000 living cells were seeded onto a 100 mm plate in their respective culture media. The plates were then incubated at 37 °C in a humidified incubator with 5% CO_2_ for 9 days. After the incubation, crystal violet stain was performed, and the living cells were indicated by the total number of stained cell clones on the 100 mm plates. At least 50 cells were required to define a colony.

### Apoptosis assay

To evaluate the effect of apoptosis induced by irrigation fluids and MMC at normothermic and hyperthermic conditions to CaB cells, Annexin V-FITC and propidium iodide (PI) (BD Pharmingen, USA) double stain was employed. In this assay, 1 × 10^6^ cells/mL suspended living cells of the 4 CaB cell lines were incubated at different irrigation fluids and MMC of different concentrations or culture media for 1 h at 37 °C and 43 °C. After incubation, cells were washed twice with PBS (Phosphate Buffered Saline), followed by Annexin V-FITC and PI staining. Fluorescent signal was immediately measured by flow cytometry (BD FACSDiva v.8.0.1, BD LSRFortessa, BD Pharmingen, USA) and data was further analysed by FlowJo software (version 10.4).

### Statistical analysis

Data are plotted as mean ± standard error of the mean (SEM) at least three independent experiments. Statistical analysis was performed by Student t-test for independent samples with equal variances. The level of significance was set at p < 0.05 as *, and p < 0.01 as **.

## Results

### The effects of normothermic and hyperthermic irrigating fluids on CaB cell lines

**Figure 1:**
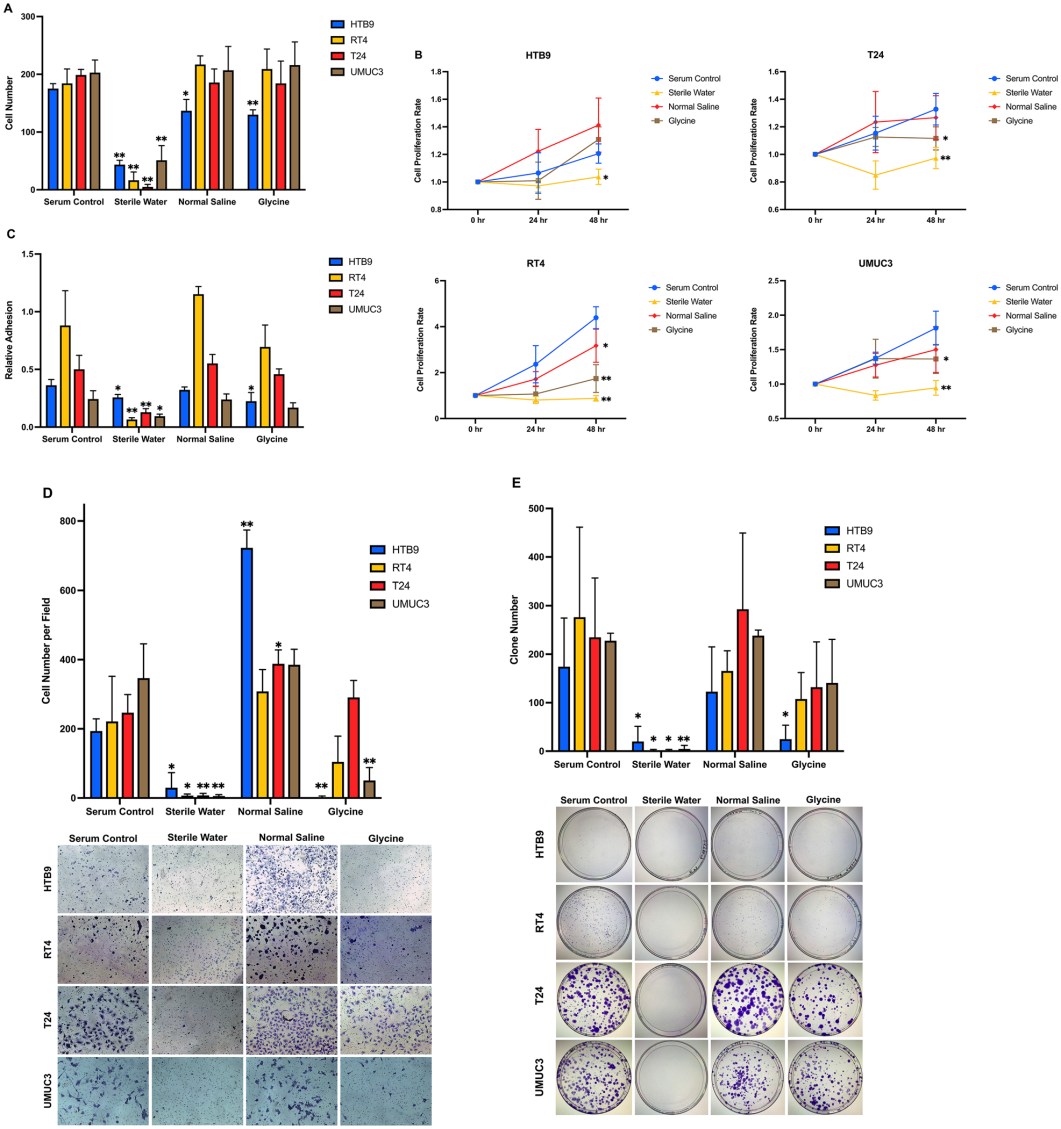
Identification of potential cytotoxicity of irrigation fluids. **(A)** Bladder cancer cell lines HTB9, RT4, T24 and UMUC3 were incubated in sterile water, normal saline or glycine for one hour. Total cell counts were quantified by trypan blue exclusion assay. **(B)** Cell proliferation rates were measured by MTT assays and presented as relative fold change to 0 h. **(C)** Cell adhesion ability were evaluated by measuring the attached cells by MTT assays and OD reading was proportional to the number of adhered cells. **(D)** Cell invasion assays were used to evaluate invasion capacity using the Matrigel invasion chambers. Cells invaded through the Matrigel layer were stained by crystal violet and counted using the ImageJ software. **(E)** Colonies formation abilities were determined by seeding 1000 living cells onto a 100 mm plate and incubated for 9 days. Colonies were then stained with crystal violet and colonies with more than 50 cells were counted. Data are presented as the mean ± SD. Different strategies were compared with serum control of the same cell line. *p < 0.05 and **p < 0.01, t test (n = 3, three independent biological replicates were performed for all the assays).

**Figure 2:**
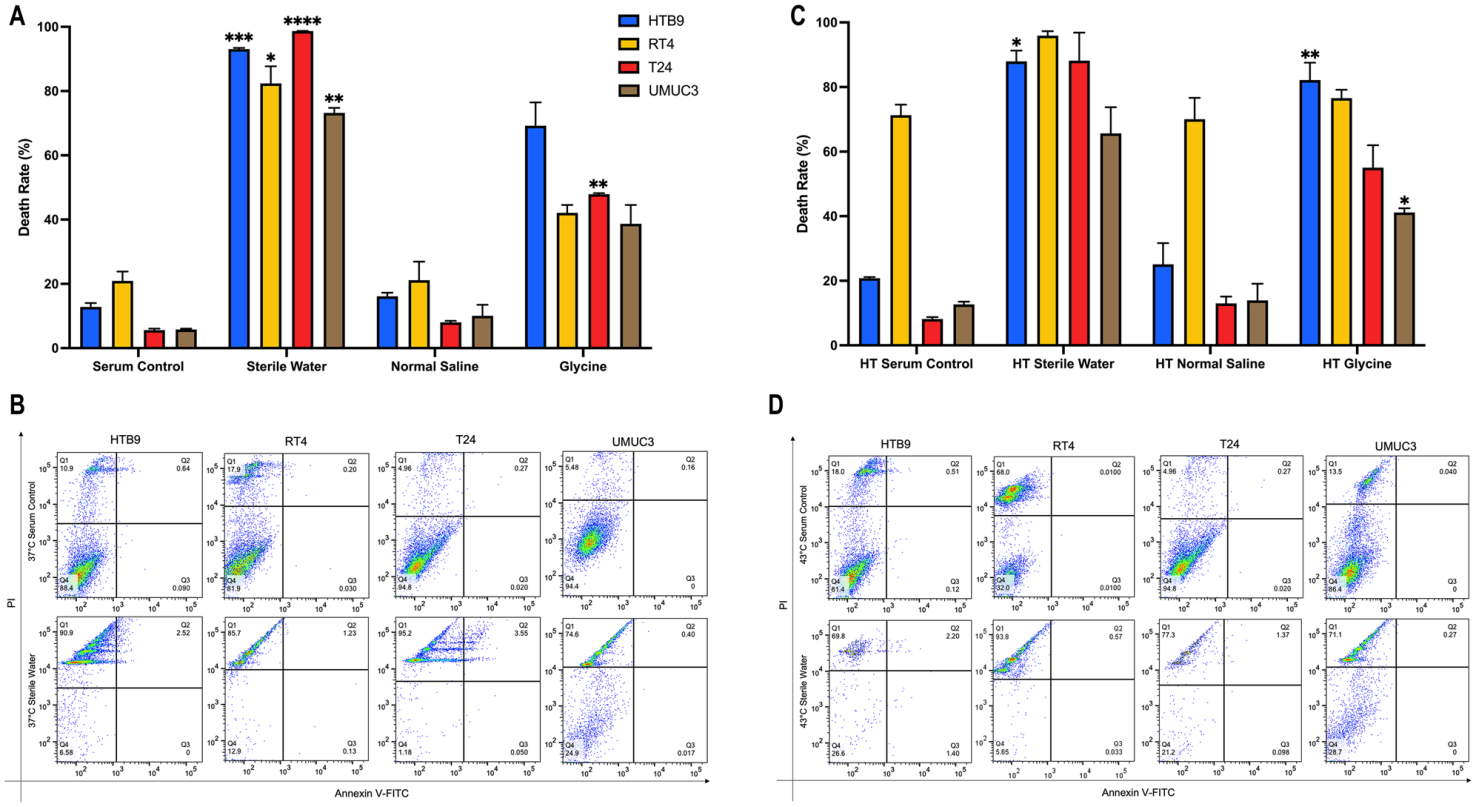
Death rates of bladder cancer cell lines analysed by flow cytometry after treating with different irrigation fluids. **(A)** HTB9, RT4, T24 and UMUC3 cell suspension were incubated in sterile water, normal saline or glycine at 37 °C for one hour. Percentages of death cells were estimated by FlowJo (version 10.4). **(B)** Representative flow cytometric charts of HTB9, RT4, T24 and UMUC3 incubated in serum control and sterile water at 37 °C for one hour. Cells at different conditions were populated at 4 quadrants. Q1-upper left: necrotic; Q2-upper right: late apoptosis; Q3-lower right: early apoptosis and Q4-lower left: viable. **(C)** HTB9, RT4, T24 and UMUC3 cell suspension were incubated in sterile water, normal saline or glycine at 43 °C for 1 h. Percentages of death cells were estimated by FlowJo (version 10.4). **(D)** Representative flow cytometric charts of HTB9, RT4, T24 and UMUC3 incubated in serum control and sterile water at 43 °C for one hour. Cells at different conditions were populated at 4 quadrants. Q1-upper left: necrotic; Q2-upper right: late apoptosis; Q3-lower right: early apoptosis and Q4-lower left: viable.Data are presented as the mean ± SD. Different strategies were compared with serum control of the same cell line. *p < 0.05 and **p < 0.01, ***p < 0.001, ****p < 0.0001, t test (n = 3, three independent biological replicates were performed for all the assays).

To evaluate the in vitro cytotoxicity of irrigation fluids, we used intra operative fluids such as water, normal saline and glycine to challenge HTB9, RT4, T24 and UMUC3. Cells were treated in suspension manner to mimic the floating of residual cancer cells inside urinary bladder. Water incubation significantly reduced total number of cells counts in the four CaB cell lines (Fig. 1A). Normal saline treatment either preserved or promoted cell growth, while water consistently inhibited cell proliferation in HTB9, RT4, T24 and UMUC3 (Fig. 1B). Concerning the adhesion assay, water suppressed adhesion in all four CaB cell lines (Fig. 1C). Similarly, water was able to suppress invasion (Fig. 1D) and colony formation (Fig. 1E) in all four CaB cell lines. In contrast, normal saline either maintained or stimulated adhesion, invasion and clonogenic ability of the four cell lines, and the effects were similar to serum control. 1.5% glycine had a toxic effect in HTB9; otherwise its effect was largely insignificant in the other three CaB cell lines. Flow cytometric analysis was also employed to study the apoptotic effects of different irrigation fluids to HTB9, RT4, T24 and UMUC3 cell suspensions. Results indicated that none of them could induce apoptosis to all the CaB cell lines. (Fig. 2A). Similar to previous assays, sterile water possessed the strongest toxic effects to the cells (Fig. 2B), following by 1.5% glycine and normal saline barely killed cells. (Supplementary Fig. S1A–D). Collectively, these results indicate that water is effective in suppressing cell growth and tumour progression, while normal saline does not introduce any adverse effects on cancer cells under normothermic condition.

**Figure 3:**
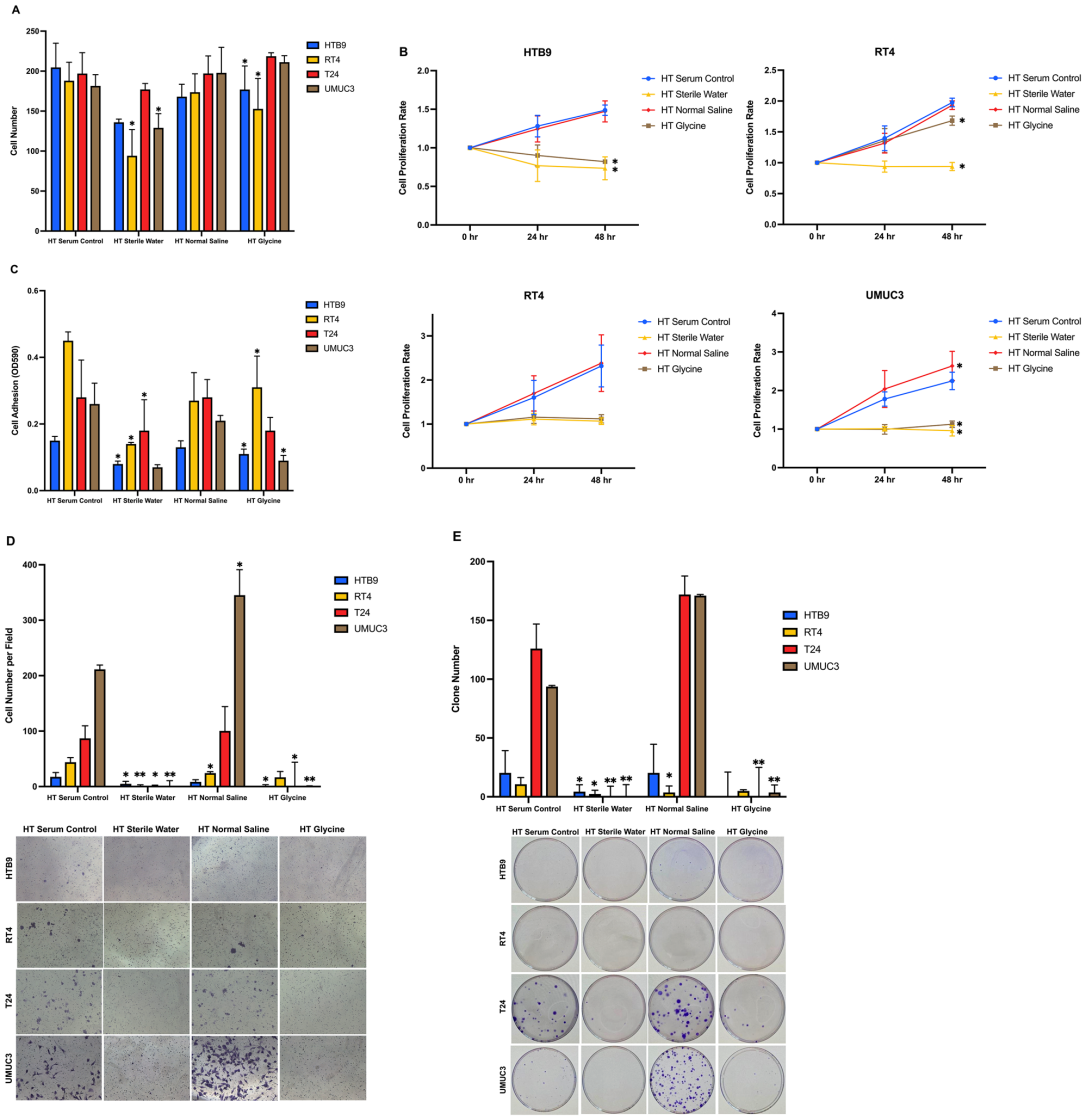
Cytotoxicity of combining hyperthermia and irrigation fluids. **(A)** HTB9, RT4, T24 and UMUC3 were incubated in sterile water, normal saline or glycine at 43 °C for one hour. Total cell counts were estimated by trypan blue exclusion assays. **(B)** Cell proliferation rates were measure by MTT assays and presented as relative fold change to 0 h. **(C)** Adhesion assay were evaluated by MTT assays as OD reading was proportional to number of adhered cells. **(D)** Invasion ability was evaluated by Matrigel gel transwell chambers. Cells with invasion ability were measured by crystal violet staining and cell counting. **(E)** Colonies formation ability was assayed by crystal violet staining and cell counting. Data are presented as mean ± SD. Different strategies were compared with serum control of the same cell line. *p < 0.05 and **p < 0.01, t test (n = 3, three independent biological replicates were performed for all the assays).

After combining with hyperthermia, we observed that water decreased total number of cells counts in the four CaB cell lines (Fig. 3A). Hyperthermic water suppressed cell proliferation in four cell lines, hyperthermic glycine inhibited cell proliferation in HTB9, T24 and UMUC3, but not RT4; while hyperthermic normal saline promoted cell growth in four cell lines as good as serum control (Fig. 3B). For cell adhesion assay, both hyperthermic water and hyperthermic glycine were able to decrease the cell adhesion ability of the four cell lines (Fig. 3C). Similarly, hyperthermic water and hyperthermic glycine reduced cells invasion (Fig. 3D) and colonies formation (Fig. 3E) in all four CaB cell lines. On the other hand, hyperthermic normal saline promoted cells invasion and clonogenic ability in UMUC3. Flow cytometry results demonstrated that hyperthermia generally induced necrosis to all cell lines by irrigation fluids (Fig. 2C). The killing effects of sterile water and 1.5% glycine were also augmented under hyperthermic conditions (Fig. 2D), meanwhile, sterile water remained as the strongest irrigation fluids in terms of cell killing (Supplementary Fig. S2A–D). In summary, our result demonstrated toxic effects of hyperthermic water and hyperthermic glycine on cell proliferation and tumour progression of all four CaB cell lines.

### The effects of normothermic and hyperthermic MMC on CaB cell lines

**Figure 4:**
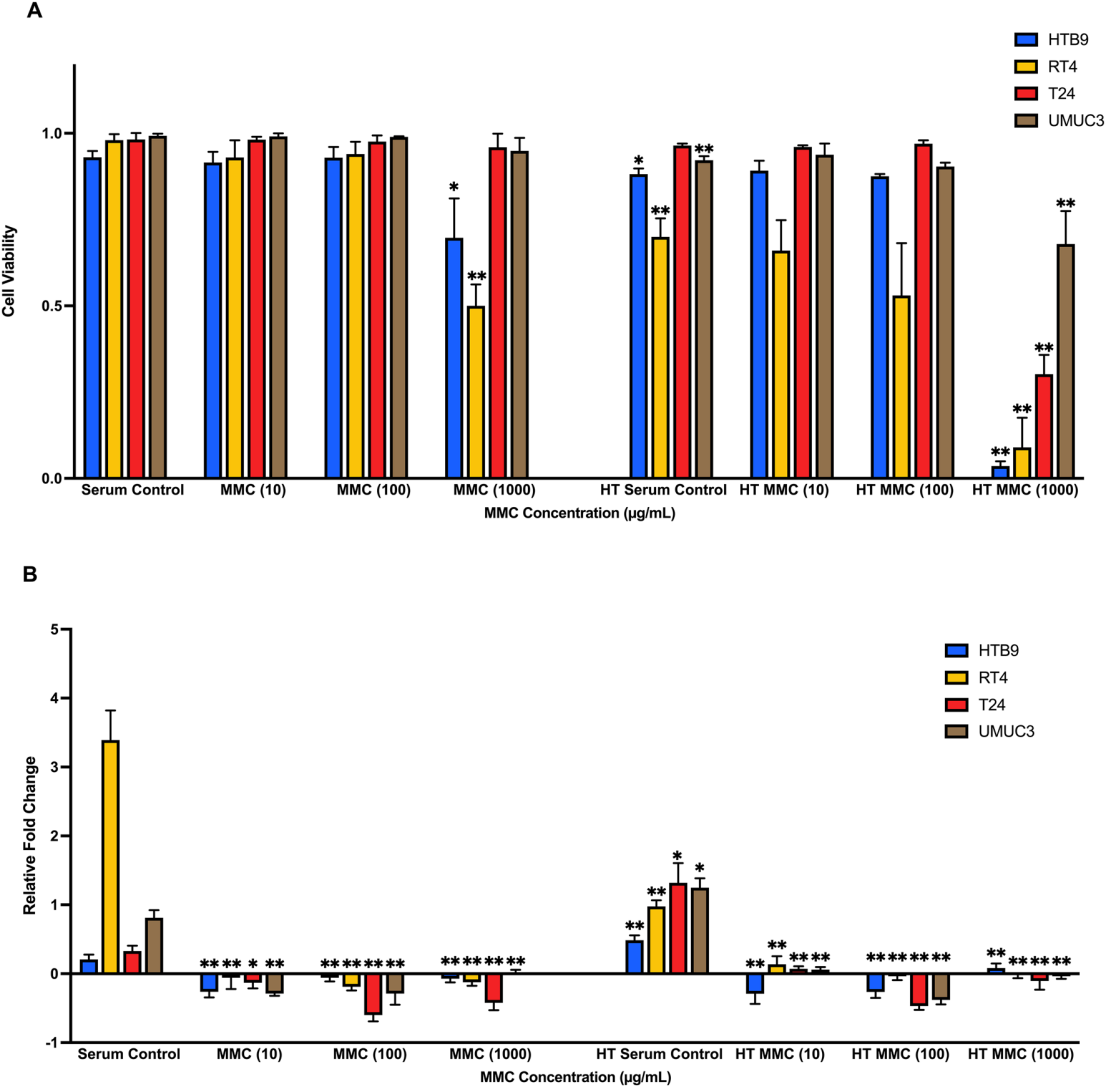

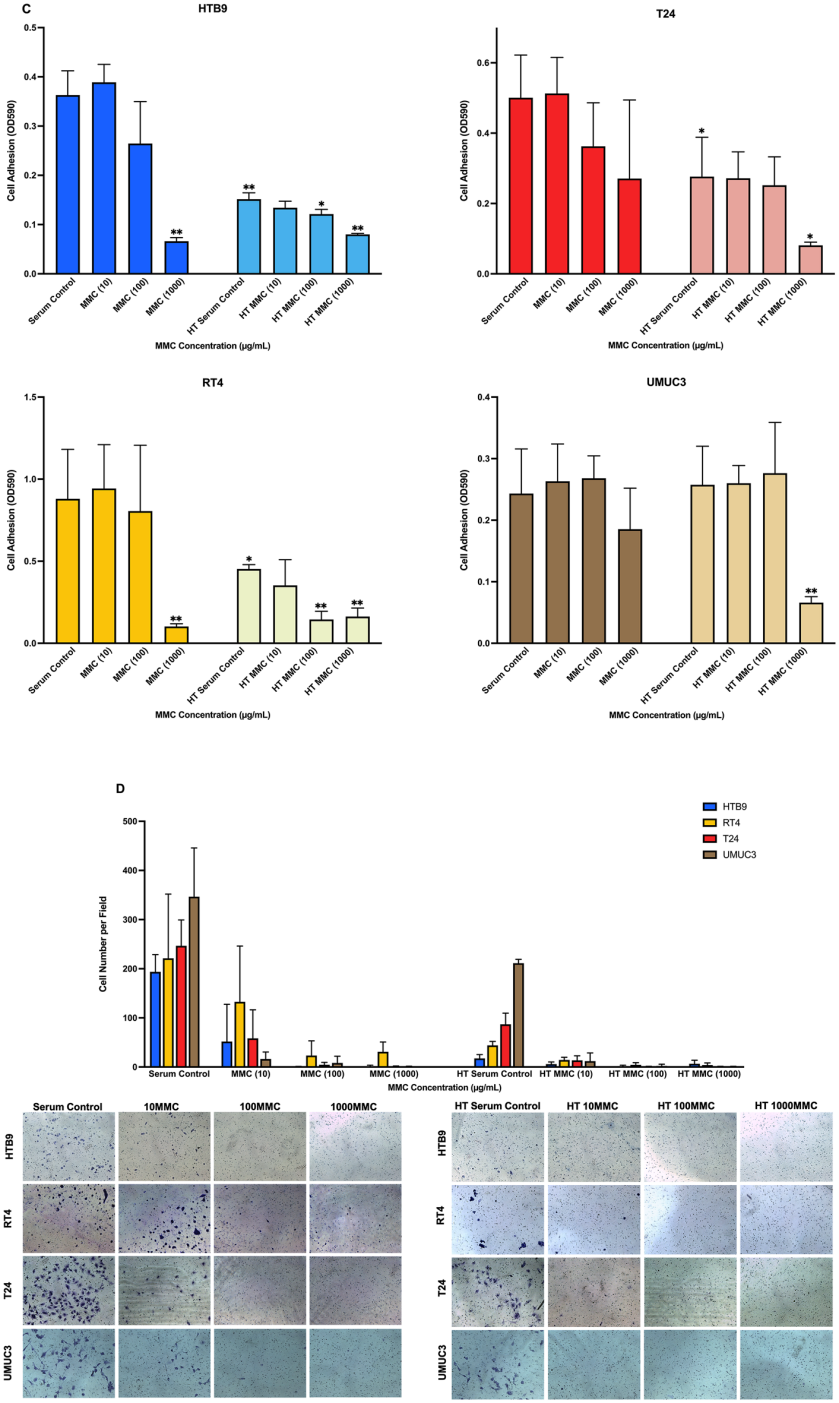

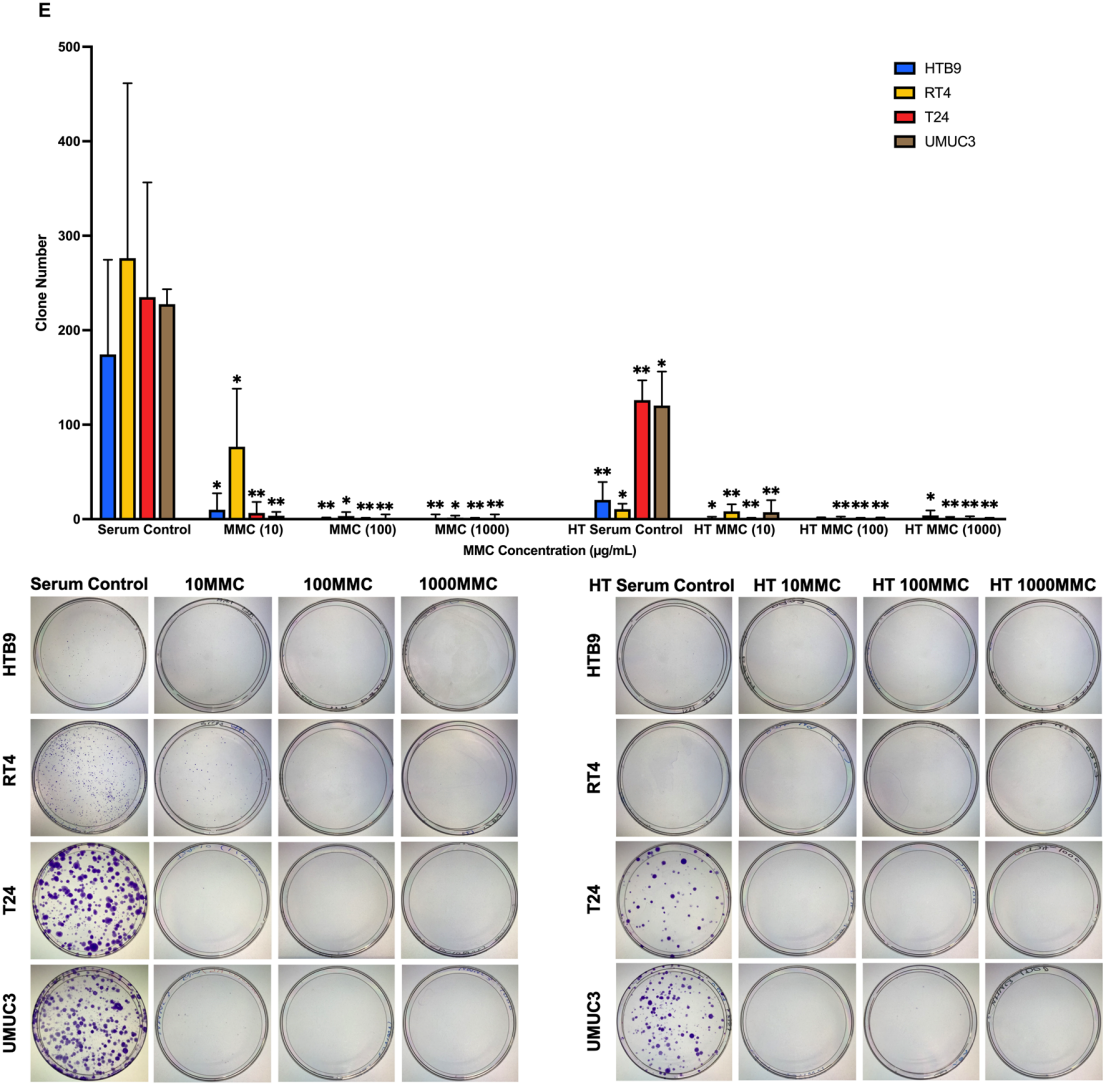
Hyperthermic Mitomycin-C markedly inhibits proliferation and tumour character of CaB cells. **(A)** Cytotoxicity of MMC was tested on bladder cancer cell lines at different concentration, 10 µg/ml, 100 µg/ml and 1000 µg/ml; at 37 °C and 43 °C. Total cell counts were calculated by trypan blue exclusion assays after drug incubation. Cell viability was evaluated and presented as percentage of living cells. **(B)** Growth suppression was measured by MTT assays and expressed as relative fold change at end point of 48 h. **(C)** Adhesion assays were measured by MTT assays and OD reading was proportional to the number of adhered cells. **(D)** Matrigel invasion abilities were evaluated by crystal violet staining and cell counting. **(E)** Colonies formation assays were performed by staining the cell clones with crystal violet. Colony with more than 50 cells was counted. Data are presented as the mean ± SD. 37 °C treatments and 43 °C serum control were compared with 37 °C serum control of the same cell line, and 43 °C treatments were compared with 43 °C serum control of the same cell line. *p < 0.05 and **p < 0.01, t test (n = 3, three independent biological replicates were performed for all the assays).

**Figure 5:**
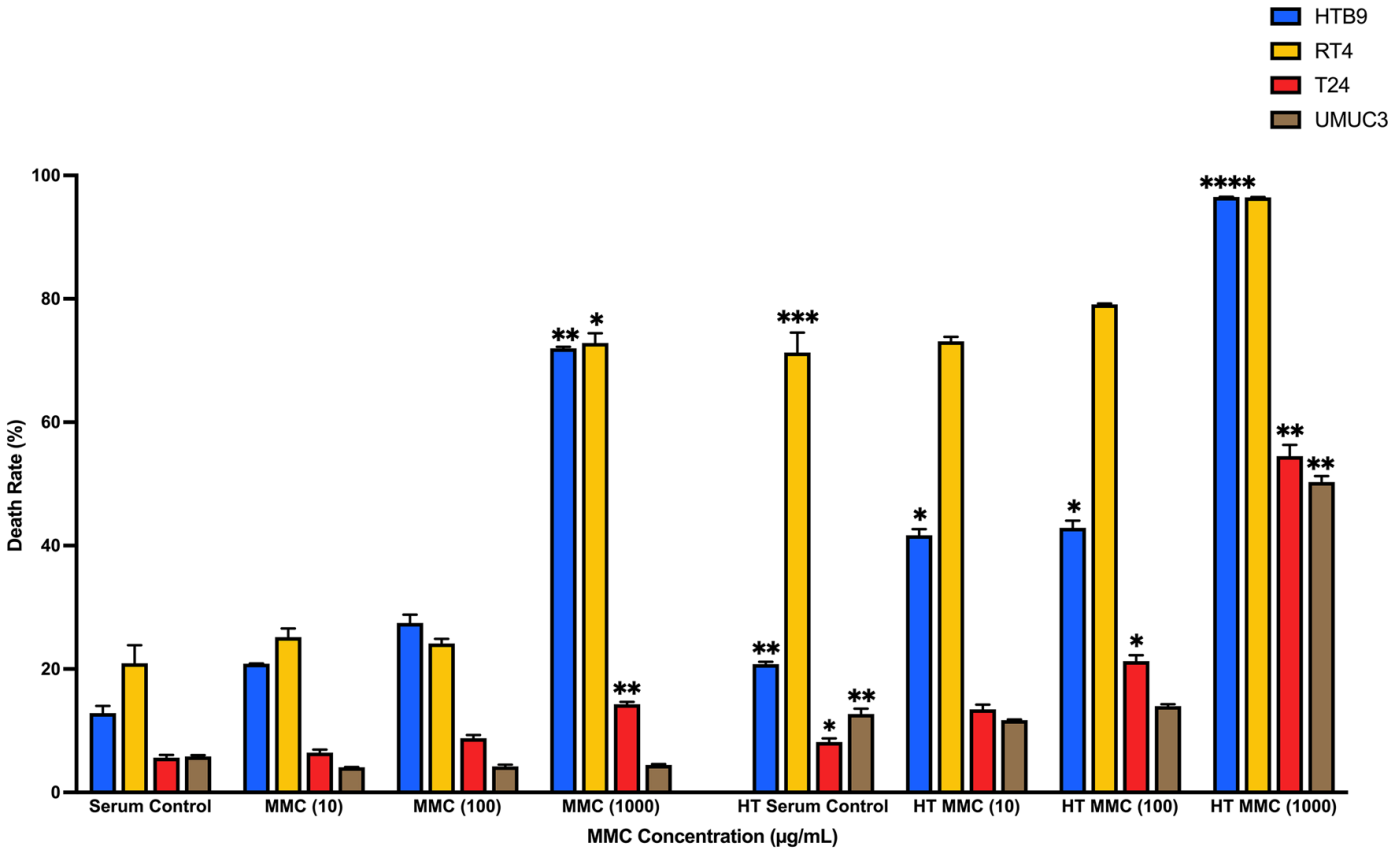
Comparison of normothermia- and hyperthermia-MMC induced cytotoxicity at different concentrations by flow cytometric analysis. HTB9, RT4, T24 and UMUC3 cell suspensions were incubated at 37 °C and 43 °C at 10 µg/ml, 100 µg/ml and 1000 µg/ml MMC. Death rates of the 4 bladder cancer cell lines were calculated by FlowJo (version 10.4). Data are presented as the mean ± SD. Different strategies were compared with serum control of the same cell line. *p < 0.05 and **p < 0.01, ***p < 0.001, ****p < 0.0001, t test (n = 3, three independent biological replicates were performed for all the assays).

To investigate the cytotoxicity response of hyperthermia drug treatment, we incubated CaB cell suspensions in 10 µg/ml, 100 µg/ml or 1000 µg/ml MMC at 37 °C or 43 °C for one hour. We observed that MMC had a dose-dependent effect; 1000 µg/ml MMC at 43 °C had the most toxic effect against all four CaB cell lines (Fig. 4A). In contrast, MMC could suppress cell proliferation regardless of concentration and temperature (Fig. 4B). At 37 °C, only 1000 µg/ml MMC could inhibit cell adhesion. Hyperthermia at 43 °C could potentiate the inhibitory effects, and 1000 µg/ml MMC at 43 °C was most effective in inhibiting adhesion in all four CaB cell lines (Fig. 4C). In addition, all concentration of MMC at different temperature significantly restricted invasion (Fig. 4D) and colonies formation (Fig. 4E) in all four CaB cell lines; 1000 µg/ml MMC at 43 °C was the most efficient regimen. As indicated by flow cytometric results, all three concentrations of MMC, as well as serum controls, could significantly induce necrosis to nearly all of the CaB cell lines upon hyperthermal incubations (Fig. 5). Cell killing effects of different concentrations of MMC, especially 1000 µg/ml, were also augmented. (Supplementary Fig. S3A–D). In summary, these results suggested that hyperthermic 1000 µg/ml MMC is the most cytotoxic reagent in CaB cell lines.

## Discussion

There are studies in recent years investigating the possibilities to reduce bladder cancer recurrence during and after surgical resection. Among all, van der Heijden et al. proposed a synergism of MMC and hyperthermia could significantly decrease proliferation rates of bladder cancer cell lines^9^. Taoka et al. showed that sterile water induced cytocidal effects on bladder cancer cell lines as well^10^. Hereby, we provided a more comprehensive study on the effects of intra-operative and post-operative environment on bladder cancer recurrence, as we believed early recurrence of bladder cancer occur as soon as during resection of tumor, when residual floating cancer cells start to reimplant onto the bladder wall. In our study, we investigated the effects of common irrigating fluids and mitomycin C, with or without hyperthermia, on bladder cancer cell lines. Four CaB cell lines in three subtypes, RT4 (luminal), HTB9/5637 (basal) and T24 and UMUC3 (non-type) were used to explore their unique cellular reaction in different culture reagents. In order to mimic residual cancer cells floating intravesically, we performed incubation of suspended cells in different irrigation fluids and we suggested this can maximally mimicked the 3D environment inside urinary bladder.

We found that water possessed the strongest toxicity to all 4 bladder cancer cell lines, in terms of proliferation, adhesion, invasion and clonal formation. Fechner et al*.* evaluated the performance of osmotic cytolysis by distilled water and mitomycin to reduce CaB recurrence. Results show that both distilled water and mitomycin led to significant cell death after 10 min to all cell lines. Nonetheless, viability of all cell lines treated with distilled water decreased to 3–5%, yet only 3 out of the 4 cell lines with survival dropped to below 5%^11^. Together with our study findings, water is shown to have strong cytotoxic effects and is promising to prevent bladder cancer cells reimplantation during surgery and recurrence. Flow cytometric results also revealed the mechanisms of cell deaths induced by sterile water which necrosis was mainly observed. To explain the phenomenon physically, water is strongly hypotonic to tumour cells, which lack cell wall protection. While surrounding by hypotonic solution, water moves into the cells from outside to balance the concentration on both side of the cells until equilibrium and equal concentration are reached. Therefore, prolong exposure of water may exert cytocidal effects of hypotonic shock to bladder cancer cell lines^12^. In extreme situation, water keeps moving into the cells, destroying normal function, bursting the cells and eventually causing cell death.

On the other hand, our study showed that normal saline possessed a weaker cytotoxic effect, therefore, could hardly prevent the reimplantation of cancer cells. Isotonic normal saline is 0.9% (9 g/L) sodium chloride, having osmolarity around 300 mOsm/L, is closely approximate to the osmolarity of sodium chloride in blood and human cells (about 290 mOsm/L). Normal saline is indeed a favorable environment for cancer cells to survive and reimplant to the bladder, which in turn may lead to tumour recurrence. For 1.5% glycine, our results showed that it had a weaker cytotoxic effect than distilled water. We observed that bladder cancer cell lines maintained proliferation in terms of tumorigenic properties upon glycine irrigation. This demonstrated that 1.5% glycine may favoured cancer cells reimplantation in bladder in vivo after resection. Nonetheless, results from flow cytometry demonstrated that glycine could induce necrosis in all CaB cell lines at both normothermic and hyperthermic conditions. Several studies indicated the usage of glycine as an irrigation fluid was toxic, a xenografts study demonstrated that intravenous injection of glycine caused more tissue damages and higher mortality^13^, suggesting that cells including but not limited to cancer cells were sensitive to glycine irrigation. These studies are reflection of our in vitro flow cytometric results of glycine on all cell lines. Besides, 1.5% glycine is a weakly ionized solution, contains 15 g/L glycine, having osmolarity of 200 mOsmol/L is slightly hypotonic to normal in vivo physiologic osmolarity condition. The weaker hypotonic property of glycine than sterile water may explain its limited cytotoxic effect to CaB cancer cell lines.

To sum up, continuous bladder irrigation with normal saline may in fact potentiate the risk of tumour reimplantation and it should be avoided as far as possible. While water is hypotonic, using it as irrigating fluid during transurethral resection surgery is not ideal due to the risk of transurethral resection syndrome. However, continuous intravesical water irrigation immediately after the surgery can be a cheap but effective method in minimising the risk of tumour reimplantation^10,12,14^. A comparison between sterile water and MMC as irrigating agents in vivo after resection was done by Bijalwan et al. Results showed that continuous bladder irrigation with sterile water after resection had comparable results to immediate intravesical MMC in terms of preventing tumour recurrence in NMIBC. Moreover, authors observed sterile water as irrigating agent could reduce adverse effects significantly when compared to MMC instillation. Besides, the recurrence-free rates were both low for sterile water-treated and MMC-treated groups though the study had a small sample size^15^.

To further investigate the cytotoxic effects induced by irrigating fluids under hyperthermia, we repeated the proliferation assay, cell adhesion assay, invasion assay and colonies formation assay on the four CaB cell lines at 43 °C. Similar to our results in the normothermic experiments, the strongest cytotoxicity was observed in water under hyperthermic condition. Water as a strong hypotonic fluid, its toxicity effect was maintained and augmented under high temperature on cell growth and other cancer properties as well as necrotic effects were also observed in four all cell lines tested. Our results also showed that hyperthermia could increase the in vitro toxicity of glycine on all cell lines. Treating cells in 43 °C glycine for one hour could greatly suppress cell adhesion, invasion and colonies formation in HTB9, RT4, T24 and UMUC3 and amplified the effects of glycine-induced necrosis. Hyperthermia alone can induce protein unfolding, ROS generation and DNA replication and repair alteration, eventually causes cell death^16^. Besides, direct cytotoxicity associated with heat exposure is related to exposure time. It was shown that one hour exposure at 43 °C induced a large extent of irreversible cytotoxicity, 30% more cell death^17^, therefore, it was expected that the cytotoxicity to CaB cell lines induced by water and glycine were at least similar or even higher under hyperthermic condition. Intriguingly, our results showed that saline under hyperthermic condition did not affect cytotoxicity as in water and glycine. In fact, upon hyperthermic saline resulted in an attenuated inhibition to 2 out of 4 CaB cell lines, T24 and UMUC3, in terms of invasion and colony formation. T24 and UMUC3 both belong to non-type CaB cell lines (Warrick 2016) under molecular characterization, and the mechanism behind this diverse response of hyperthermic normal saline to T24 and UMUC3 is uncertain and need further examination.

Intravesical maintenance chemotherapy is a standard treatment for patients with NMIBC. Although it shows a lower rate of adverse events, its treatment efficacy has been proven to be inferior to intravesical BCG therapy, especially for high-risk NMIBC patients. In recent years, people have developed device-assisted technology which can enhance chemotherapy efficiency. MMC is a naturally occurring bio-reductive alkylating agent that attacks and destroys cancerous cells, and the standard clinical concentration is 1000 µg/ml in final volume 20 ml to be instilled into bladder for an hour incubation. However, we found that 1000 µg/ml MMC under normothermic condition could not significantly reduce cell adhesion ability of non-typing cells, T24 and UMUC3. Cell adhesion plays a pivotal role in cancer progression and metastasis. Hyperthermia has been shown to boost the efficacy of cytotoxic agent treatments. Malignant cancerous cells are also more heat sensitive than normal cells or tissues^9^. From our results, we demonstrated that hyperthermia at 43 °C in synergy with MMC resulted in a stronger cytotoxic effect to all 4 CaB cancer cell lines than MMC treatment in 37 °C. On the other hand, we observed MMC concentration of 1000 µg/mL exhibited the strongest inhibition in proliferation and cancer properties of the four CaB cell lines. Nonetheless, the mode of cell death induced by one hour incubation of MMC at different concentrations were mainly occurred as necrosis but not apoptosis. Several previous studies reported the in vitro and in vivo effects of MMC at different concentrations^18–20^. Yoshida et al. observed DNA fragmentation, which is hallmark of apoptosis, was not surfaced upon addition of MMC to their CaB-derived spheroids, suggesting apoptosis did not occur, therefore, the authors concluded that high dose MMC (1 mg/ml) did not induce apoptosis, but necrosis and autophagy^20^. One explanation to our observations is that tumour cells may uptake higher amount of MMC and are less resistant to the drugs under hyperthermia treatment. Besides, MMC may have increase cytotoxic activities under higher temperatures, as heat can destroy cell membrane fluidity, weaken or denature cellular proteins, losing protein function will eventually causing decrease in proliferation and increase in apoptosis of cancer cells. Moreover, another signature of our results was the cell death induced by irrigation fluids or MMC at normothermic and hyperthermic conditions was mainly necrosis. Qian et al. evaluated heat stress-induced necrosis rates at rat cardiomyocyte. Results indicated a transient exposure at 43 °C (< 3 h) mainly induced necrotic cell death and apoptosis surfaced only after 4 h exposure^21^, therefore, we suggested other types of cell death such as necrosis and autophagy were induced upon heat stress and treating cells with sterile water, 1.5% glycine and high dose MMC. Our results supported the use of hyperthermic chemotherapy in the clinical setting to optimise the oncological outcomes of NMINC following TURBT.

## Limitations

Our study investigated the intra-operative and post-operative intravesical environment in preventing CaB cell lines reimplantation. The cytotoxic effects of water as an irrigation agent to CaB cancer cell lines, and the synergistic effect of hyperthermia and MMC in inhibiting proliferation of cancer cells in vitro were explored. The results are promising in translating into clinical studies and applications. Nonetheless, there are also some limitations in our study. First of all, our results on the in vitro setting might not be replicated in the in vivo setting. Further investigations using animal models could be considered before translating into clinical practice^11^. Secondly, the mechanisms underlying the synergistic effects of hyperthermia and MMC in different cancer cell lines require further investigations. The synergistic effects may vary depending on the concentrations of MMC and the type of cell lines being tested. In our results, UMUC-3 exhibited the greatest drug resistance towards MMC, even under hyperthermia treatment, in terms of immediate toxicity effects. Thirdly, although the CaB cell lines utilized represented different stages and grades of bladder tumour, they are still limited in mimicking the in vivo conditions. For example, cell lines cannot illustrate the interactions between bladder tumour and surrounding cells. Besides, morphology and physiological parameters may alter upon prolong culture of cancer cells on plastic surface. Therefore, more advance models should be considered before applying our results in clinical practices. For example, 3D organoid cultures serve as a better to mimic cell–cell interactions and in vivo human environment. Further studies can be applied on immortalized cell line derived-organoid or even patient primary tumour-derived organoid models to validate our results.

## Conclusion

Urinary bladder cancer is a common cancer in worldwide. Although TURBT is currently the standard to resect NMIBC tumour, it accompanies with high recurrence rate due to residual cancer cell reimplantation. Hereby, we investigated intra-operative and post-operative environments in reducing bladder cancer recurrence. In our study, we investigated the cytotoxic effects of different irrigating fluids to CaB cell lines, in terms of tumorigenic properties. Our results demonstrated that water is the most cytotoxic agents to CaB cell lines when compared to glycine and saline, which could only barely kill cancer cells or even maintained their tumorigenic properties. We further investigated the synergic effects of hyperthermia and irrigating agents as well as MMC. Our results demonstrated that water still possessed the highest cytotoxic effects to CaB cell lines under hyperthermic condition. Besides, synergism among hyperthermia and MMC also exhibited high efficacy in killing cancer cells. Our results are promising in studying how different conditions, during resection and after surgery, may contributed to bladder cancer recurrence. Nonetheless, further experiments should be carried out, for instance, xenograft models, before translating our results into clinical settings.

## Supplementary Information

Supplementary Figure S1.

Supplementary Figure S2.

Supplementary Figure S3.

Supplementary Legends.
