# Supplementary material for: In vitro assessment of intra-operative and post-operative environment in reducing bladder cancer recurrence
Source: Sci Rep. 2022 Jan 7;12:22. doi: 10.1038/s41598-021-04035-8 (PMC8741939; doi:10.1038/s41598-021-04035-8)
Supplement: Supplementary file 4 — Supplementary Legends. [file 41598_2021_4035_MOESM4_ESM.docx]

Supplementary Fig. S1A-D. Representative flow cytometric results of (A) HTB9, (B) RT4, (C) T24 and (D) UMUC3 upon incubating with serum control, sterile water, normal saline, 1.5% glycine at 37°C. Cells at different conditions were populated at 4 quadrants. Q1-upper left: necrotic; Q2-upper right: late apoptosis; Q3-lower right: early apoptosis and Q4-lower left: viable.

Supplementary Fig. S2A-D. Representative flow cytometric results of (A) HTB9, (B) RT4, (C) T24 and (D) UMUC3 upon incubating with serum control, sterile water, normal saline, 1.5% glycine at 43°C. Cells at different conditions were populated at 4 quadrants. Q1-upper left: necrotic; Q2-upper right: late apoptosis; Q3-lower right: early apoptosis and Q4-lower left: viable.

Supplementary Fig. S3A-D. Comparison of representative flow cytometric results of (A) HTB9, (B) RT4, (C) T24 and (D) UMUC3 upon incubating with serum control, 10µg/ml MMC, 100µg/ml MMC and 1000µg/ml at 37°C versus 43°C. Cells at different conditions were populated at 4 quadrants. Q1-upper left: necrotic; Q2-upper right: late apoptosis; Q3-lower right: early apoptosis and Q4-lower left: viable.
